# Metabolomics analysis of the soapberry (*Sapindus mukorossi* Gaertn.) pericarp during fruit development and ripening based on UHPLC-HRMS

**DOI:** 10.1038/s41598-021-91143-0

**Published:** 2021-06-02

**Authors:** Yuanyuan Xu, Yuan Gao, Zhong Chen, Guochun Zhao, Jiming Liu, Xin Wang, Shilun Gao, Duanguang Zhang, Liming Jia

**Affiliations:** 1 Key Laboratory of Silviculture and Conservation of the Ministry of Education, College of Forestry, Beijing Forestry University, 35 E Qinghua Road, Beijing, 100083 China; 2 National Energy R&D Center for Non-Food Biamass, Beijing Forestry University, Beijing, 100083 China; 3 National Innovation Alliance of Sapindus Industry, Beijing Forestry University, Beijing, 100083 China; 4 Planning and Design Institute of Forest Products Industry, National Forestry and Grassland Administration, Beijing, 100010 China; 5 Beijing Advanced Innovation Center for Tree Breeding by Molecular Design, Beijing Forestry University, Beijing, 100083 China; 6 Yuanhua Forestry Biological Technology Co., Ltd., Sanming, 650216 Fujian China

**Keywords:** Biochemistry, Physiology, Plant sciences, Chemistry

## Abstract

Soapberry (*Sapindus mukorossi* Gaertn.) is a multi-functional tree with widespread application in toiletries, biomedicine, biomass energy, and landscaping. The pericarp of soapberry can be used as a medicine or detergent. However, there is currently no systematic study on the chemical constituents of soapberry pericarp during fruit development and ripening, and the dynamic changes in these constituents still unclear. In this study, a non-targeted metabolomics approach using ultra-high performance liquid chromatography-high resolution mass spectrometry (UHPLC-HRMS) was used to comprehensively profile the variations in metabolites in the soapberry pericarp at eight fruit growth stages. The metabolome coverage of UHPLC-HRMS on a HILIC column was higher than that of a C18 column. A total of 111 metabolites were putatively annotated. Principal component analysis and hierarchical clustering analysis of pericarp metabolic composition revealed clear metabolic shifts from early (S1–S2) to late (S3–S5) development stages to fruit ripening stages (S6–S8). Furthermore, pairwise comparison identified 57 differential metabolites that were involved in 18 KEGG pathways. Early fruit development stages (S1–S2) were characterized by high levels of key fatty acids, nucleotides, organic acids, and phosphorylated intermediates, whereas fruit ripening stages (S6–S8) were characterized by high contents of bioactive and valuable metabolites, such as troxipide, vorinostat, furamizole, alpha-tocopherol quinone, luteolin, and sucrose. S8 (fully developed and mature stage) was the most suitable stage for fruit harvesting to utilize the pericarp. To the best of our knowledge, this was the first metabolomics study of the soapberry pericarp during whole fruit growth. The results could offer valuable information for harvesting, processing, and application of soapberry pericarp, as well as highlight the metabolites that could mediate the biological activity or properties of this medicinal plant.

## Introduction

Soapberry (*Sapindus mukorossi* Gaertn., Sapindaceae, *Sapindus*) is an economic tree with widespread application in toiletries, biomedicine, biomass energy, and landscaping. It is mainly distributed in the south of the Yangtze River in China, the Indochina Peninsula, India, and Japan^1^. Soapberry is a traditional medicinal plant in China, where the fruit is the main part utilized. According to the Compendium of Materia Medica, an ancient Chinese pharmaceutical book, soapberry pericarp can be used to wash the hair and face to cure dandruff and freckles, respectively^2^. Modern pharmacological studies have also shown that soapberry pericarp has anti-inflammatory, anti-tumor, anti-bacterial, anti-viral, hepatoprotective, insecticidal, and other bioactivities^3–7^. It is also rich in saponins, has good non-ionic surface activity, high foaming property, and strong decontamination ability that can replace commonly used raw materials for detergent production^8^. Previous studies have reported that the main chemical constituents of the soapberry pericarp are terpenoids (especially triterpenoid saponins and sesquiterpenoid glycosides), phenylpropanoids, steroids, and saccharides^4,9–12^. However, few studies have reported the chemical characteristics of the pericarp during the fruit growth of soapberry.

We divided the fruit growth of soapberry into two main stages, fruit development and fruit ripening, based on the BBCH (Biologische Bundesanstalt, Bundessortenamt und CHemische Industrie) scale^13^. Fruit development and fruit ripening were further divided into five and three detailed growth stages, respectively. Generally, the optimal harvest time of a fruit influences the quality of its chemical properties and bioactivity^14^. The chemical components of soapberry pericarp may vary during different fruit growth stages; thus, to ensure its medicinal quality, it is important that soapberry is harvested at the right time.

Metabolomics is a powerful platform for the comprehensive profiling of a series of small-molecule metabolites (MV < 1000)^15^. Comparative metabolomic research allows for the understanding of comprehensive metabolite variation in a biological system^16^. Recently, metabolomic approaches related to fruit development and ripening have been used in several plants such as pineapple^17^, peach^18^, banana^19^, tomato^20^, *Lonicera caerulea*^14^, and grape^21^. Ultra-high performance liquid chromatography-high resolution mass spectrometry (UHPLC-HRMS) is a powerful tool for metabolic profiling that can provide accurate, sensitive, and reproducible measurements of a large number of small molecule compounds^15,22^. Moreover, efficient chromatographic separation is essential for metabolomics because it can separate isomers and reduce ion suppression^23,24^. Reverse phase (RP) liquid chromatography (RPLC) is suitable for separating nonpolar and medium polar compounds, whereas hydrophilic interaction liquid chromatography (HILIC) is suitable for separating highly polar compounds^22,25,26^. Hence, the combination of RPLC and HILIC can optimize the separation of compounds and expand the coverage of the metabolome.

To date, analysis of the chemical composition of soapberry pericarp at different development and ripening stages have not been reported. In this study, we compared the metabolic profiles of soapberry pericarp during fruit development and ripening using UHPLC-HRMS on a C18 column and a HILIC column to evaluate the pericarp quality from a metabolomics perspective. The differential metabolites were screened at different fruit development stages, and their variations and biological functions were analyzed. To the best of our knowledge, this is the first time that the metabolites in the soapberry pericarp was profiled during the whole fruit growth.

## Materials and methods

All experimental research and field studies on plants, including the collection of plant material in this study, had complied with relevant institutional, national, and international guidelines and legislation.

### Plant materials

**Figure 1:**
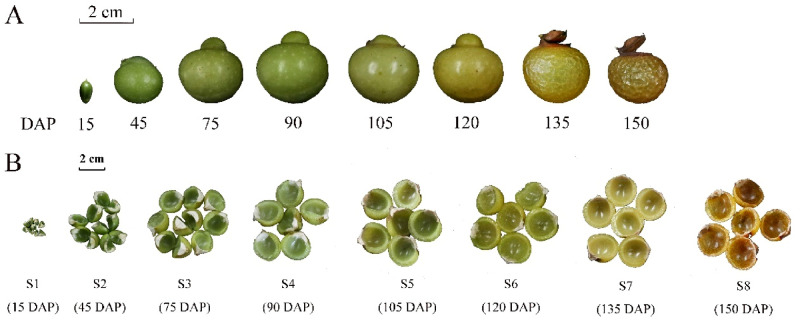
Soapberry sample from eight different fruit growth stages. This classification is based on the fruit development and fruit ripening: S1, early ovary development (15 DAP); S2, 30% of the biggest fruit size (45 DAP); S3, 70% of the biggest fruit size (75 DAP); S4, 80% of the biggest fruit size (90 DAP); S5, 90% of the biggest fruit size (105 DAP); S6, beginning of maturity (120 DAP); S7, great change in pericarp (135 DAP); S8, fully developed and mature (150 DAP). (**A**) Soapberry fruit; (**B**) soapberry pericarp.

Three soapberry plants, with 10 a, 6.5 m average tree height, 13.5 cm average diameter at breast height (DBH), and 20 kg average annual output, were obtained from an orchard located in Jianning County, Fujian Province, China (26°49′ N latitude, 116°52′ E longitude, 300 m above sea level), with the permission of managers and professionals. Moreover, this study did not involve endangered or protected species. According to our previous investigation^13^, the soapberry fruits were sampled at eight main time points from June to November 2018, including five main stages of fruit development [early ovary development (S1), approximately 15 days after pollination (DAP); 30% of the biggest fruit size (S2), 45 DAP; 70% of the biggest fruit size (S3), 75 DAP; 80% of the biggest fruit size (S4), 90 DAP; 90% of the biggest fruit size (S5), 105 DAP] and three main stages of fruit ripening [beginning of maturity (S6), 120 DAP; great change in pericarp (S7), 135 DAP; fully developed and mature (S8), 150 DAP]^13^ (Fig. 1). Three biological replicates were taken at each time point, comprising a total of 24 samples. Fruits were randomly picked from the east, south, west, and north directions of the middle and upper parts of the crown of the trees. The number of fruits picked per tree was determined by their size. Each biological replicate was comprised of fruits randomly extracted from the pool of soapberry fruits collected from each tree. After collection, the pericarp and the seed were separated, and the pericarps were immediately frozen in liquid nitrogen and stored at − 80 °C. The pericarps were dried using a vacuum freeze dryer (LGJ-10, Beijing Songyuan Huaxing Biotechnology Co., Ltd., Beijing, China) and then ground into a uniform powder using a ball mill (MM400, Retsch, Germany).

### Metabolite profiling

#### Sample preparation

The sample (30 mg) was extracted with 0.27 mL methanol/H_2_O (75:25, v/v) via ultrasonic treatment for 30 min. The solution was centrifuged at 14,000×*g* for 10 min at 4 °C and then the supernatant was evaporated to dryness under vacuum. The samples were reconstituted in 0.15 mL of either acetonitrile (ACN)/H_2_O/formic acid (FA) (50:50:0.1, v/v/v) or ACN/water (60:40, v/v) containing 1.5 ppm of sulfadimethoxine, l-valine-1-13C and pyruvic-1-13C acid as internal standards for subsequent liquid chromatography–mass spectrometry (LC–MS) analyses on RP and HILIC separation, respectively. To evaluate the repeatability of the analytical system, a quality control (QC) sample was prepared by mixing equal amounts of all samples.

#### LC–MS analysis of C18 column

Chromatographic separation was performed on a Vanquish UHPLC system with an Accucore Vanquish C18+ column (100 × 2.1 mm i.d., 1.5 μm) coupled to a Q Exactive™ Hybrid Quadrupole-Orbitrap High Resolution Mass Spectrometer (Thermo Fisher Scientific, San Jose, CA, USA). The mobile phase consisted of (A) water/0.1% FA and (B) ACN/0.1%FA. The gradient was as follows: 0.0–2.0 min (0% B), 2.0–4.0 min (0–15% B), 4.0–14.0 min (15–32% B), 14.0–19.0 min (32–50% B), 19.0–19.1 min (50–100% B), 19.1–21.0 min (100% B), 21.0–21.1 min (100–0% B), and then initial conditions were maintained for 5 min (21.1–25.0 min at 0% B) to equilibrate the column. The flow rate was 320 μL/min, and the injection volume was set to 2 μL. To avoid possible bias, the sequence of injections was randomized, with a QC run after the injection of five samples, to normalize the data for analysis. The conditions for mass spectrometry have been previously described^27^. In brief, nitrogen as the sheath, auxiliary, and sweep gas was set at 50, 8, and 1 U, respectively. Data were acquired at a resolving power of 120,000 (at m/z 200), with automatic gain control target of 3 × 10^6^ ions, 100 ms maximum injection time, 70–1050 m*/z* scan range, 3.50 kV spray voltage, and 275 °C capillary temperature. ESI−/+ data-dependent MS/MS spectra were generated for the QC samples and were used for identification purposes. MS/MS data were acquired with a full scan followed by the top 15 MS/MS scans, with a resolving power of 15,000 (at m/z 200), automatic gain control target at 1 × 10^5^ ions, maximum injection time of 50 ms, and 0.4 m/z isolation window, as well as normalized collisional energy (NCE) at 20, 30, and 40. The acquired raw files were processed using Compound Discoverer 2.1. The software parameters were 5 ppm mass tolerance for the adaptive curve model, and 0.5 min maximum shift for alignment. The software parameters for detecting unknown compounds were as follows: mass tolerance for detection of 5 ppm, intensity tolerance of 30%, signal-to-noise threshold of 3, and minimum peak intensity of 2 × 10^6^. Peaks with a coverage value > 50% and an RSD value < 30% for the QC injections were retained for subsequent analysis^28^. Metabolite annotation was performed based on a comparison of accurate mass, retention time, and MS^2^ fragments with in-house metabolite database and metabolite online databases mzCloud (https://www.mzcloud.org/) and ChemSpider (http://www.chemspider.com/). For one detected peak, one to ten various metabolite candidates were obtained, and the commonly found metabolites in plants that showed the same name from at least two databases mentioned above were selected^29^.

#### LC–MS analysis of HILIC column

Chromatographic separation on a Vanquish UHPLC system, with a SeQuant ZIC pHILIC column (150 × 2.1 mm i.d., 5 μm) coupled to a QE-HF mass spectrometer (Thermo Fisher Scientific, San Jose, CA, USA)^27^, was performed with a mobile phase that consisted of (A) 10 mmol/L AcONH_4_ in water (pH 9.8) and (B) ACN. The gradient was as follows: 0.0–1.0 min (90% B), 1.0–15.0 min (30% B), 15.0–18.0 min (30% B), 18.0–19.0 min (90% B), 19.0–29.0 min (90% B), and then initial conditions were maintained for 5 min to equilibrate the column. The flow rate was 250 μL/min, and the injection volumes were set to 2 μL. All samples, including a QC sample that contained equal amounts of each sample, were analyzed by negative and positive electrospray ionization in full scan MS mode with a mass range of 70–1050 m/z, while the rest setting of the MS parameters was the same as those for C18. The acquired raw files were processed using Compound Discoverer 2.1, as previously described.

#### Data analysis

A three-dimensional data matrix, which included the metabolite name (putatively annotated by UHPLC-HRMS), sample information (three biological repeats for each sample), and raw abundance (peak area for each putatively annotated metabolite) was generated (Table S1). Raw data were subjected to three categories of normalization: normalization by median, log transformation, and Pareto scaling. The R statistical environment was used to perform univariate analysis (fold change analysis, Student’s *t* test) and hierarchical clustering analysis (Pearson correlation distances, Ward’s clustering algorithm). SIMCA 14.1 software (Umetrics, Umea, Sweden) was used to perform unsupervised principal component analysis (PCA) and supervised orthogonal projection to latent structure-discriminant analysis (OPLS-DA). Metabolites with a variable importance projection (VIP) > 1, false discovery rate (FDR) < 0.05, and fold change (FC) > 3 or < 0.33 were determined as more important metabolites (differential metabolites) during the soapberry pericarp development and ripening. These differential metabolites were then uploaded to the MetaboAnalyst 4.0 (http://www.metaboanalyst.ca/) platform for Kyoto Encyclopedia of Genes and Genomes (KEGG) metabolic pathway analysis^30,31^.

## Results

### Metabolic profiles

UHPLC-HRMS-based untargeted metabolomic approaches were used for metabolic profiling of the soapberry pericarp at eight different growth stages. After pre-processing, 1790 features were extracted from the UHPLC-HRMS on the C18 column in negative mode, and 13,000 features (5000 in negative mode and 8000 in positive mode) were extracted from the HILIC column. Unfortunately, no metabolite was extracted using the C18 column in positive mode. Compared with the in-house database and the online databases, 56 metabolites were putatively identified in the negative ion mode on the C18 column. Of these, 19 were putatively annotated, and 37 metabolites were assigned their putative chemical formula. On the HILIC column, in the negative ion mode, 265 metabolites were putatively identified, with 88 being putatively annotated and 177 metabolites being assigned their putative chemical formula, whereas in the positive ion mode, 53 metabolites were putatively identified, 28 of which were putatively annotated and 25 were assigned their putative chemical formula.

**Figure 2:**
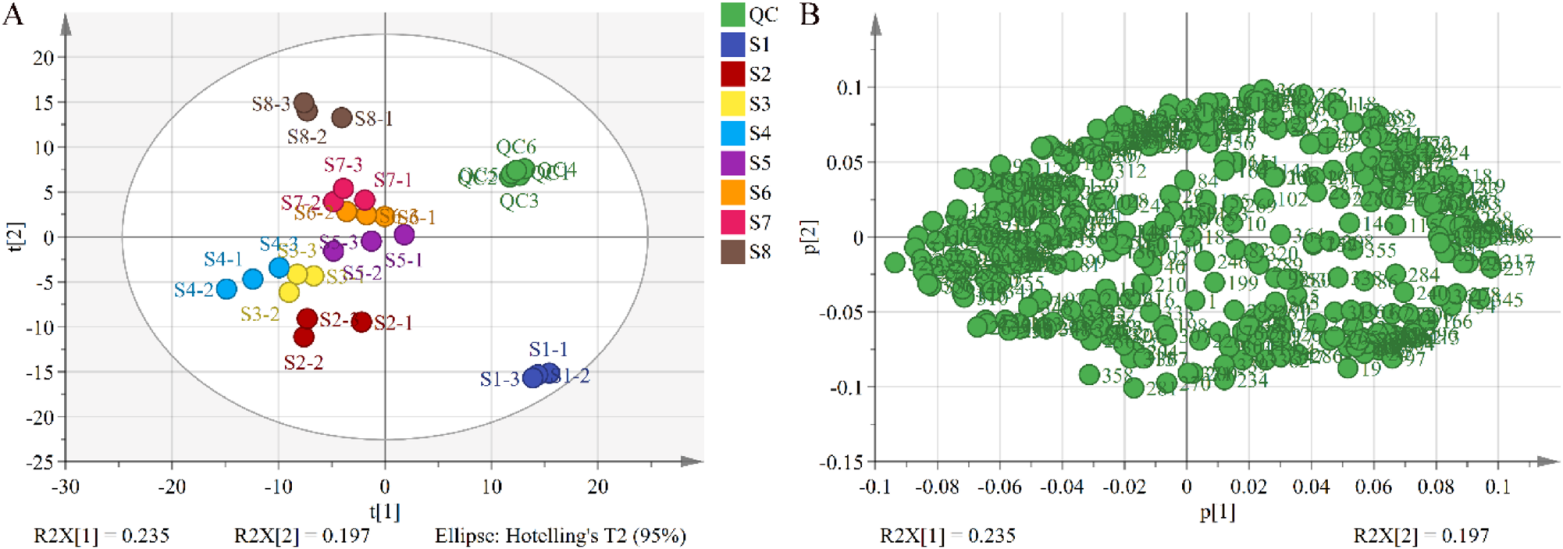
PCA result from soapberry pericarp from different fruit growth stages. Variables used for PCA were 374 putatively identified metabolites by UHPLC-HRMS on the C18 column and HILIC column. Data was auto scaled prior to PCA. (**A**) Scores plot; (**B**) loading plot.

To assess the similarities and differences in the metabolite profiles of the 24 soapberry pericarp samples at eight different growth stages, unsupervised PCA was performed, and according to the score plot (Fig. 2A), QCs were clustered crowdedly, indicating that the experimental method was reliable and the instrument was stable. In addition, all 24 samples were within the 95% confidence regions, and the eight groups were clearly separated, indicating that there were significant differences among the samples. The loading plot in Fig. 2B shows the metabolite accumulation in the soapberry pericarp at eight different growth stages. Excluding the common metabolites, 111 metabolites were putatively annotated (Table S2), including 34 amino acids and their derivatives, 12 organic acids, 10 fatty acids, nine amines, six flavonoids, six nucleotides and their derivatives, five alkaloids, four carbohydrates, four terpenoids, three vitamins, three phosphorylated intermediates, two phenylpropanoids, and 13 other metabolites.

**Figure 3:**
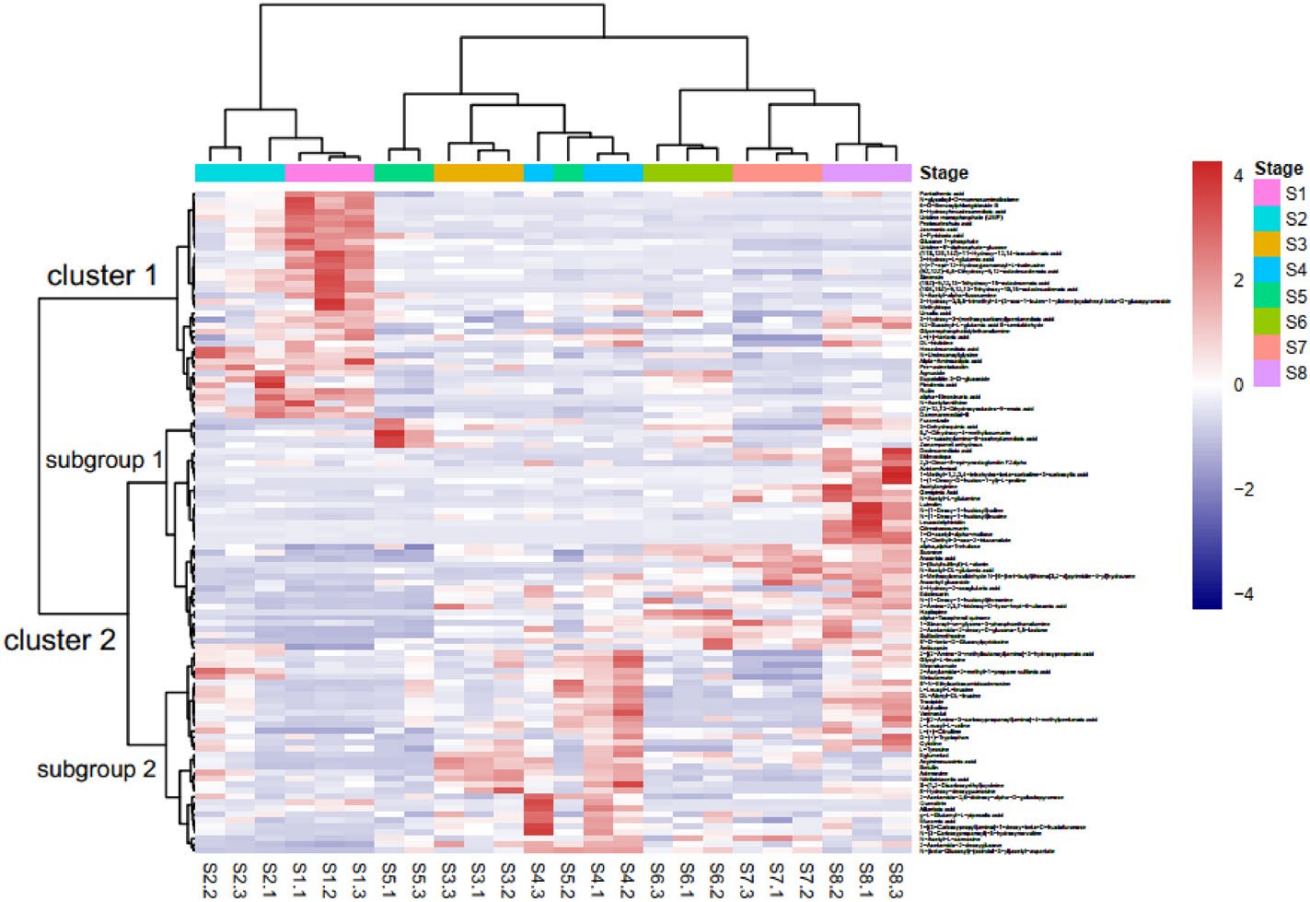
Heatmap of the 111 putatively annotated metabolites.

Furthermore, the abundance of 111 putatively annotated metabolites was analyzed by hierarchical clustering analysis (HCA) with a heat map, and results showed that the metabolites could be divided into two clusters based on their abundance during different stages. Stages S1 and S2 were classified into one cluster, while the others (stages S3–S8) were in another cluster (Fig. 3). Upon further examination, we observed that stages S3–S8 were divided into two subclusters: S3–S5 and S6–S8. Similarly, the thermogram was also divided into two clusters based on metabolite changes during fruit development. Cluster 1 included 38 metabolites (nine amino acids and their derivatives, five organic acids, eight fatty acids, one amine, three flavonoids, two nucleotides and their derivatives, three terpenoids, one vitamin, two phosphorylated intermediates, and four other metabolites), and the levels of which were highest during stage S1 and then gradually decreased. Cluster 2 could be further divided into two subgroups; subgroup 1 contained 39 metabolites (nine amino acids and their derivatives, five organic acids, two fatty acids, one amine, two flavonoids, five alkaloids, three carbohydrates, two vitamins, one phosphorylated intermediate, two phenylpropanoids, and seven other metabolites), which gradually accumulated as the fruit developed, reaching the highest at stage S8; whereas, subgroup 2 had 34 metabolites (16 amino acids and their derivatives, two organic acids, seven amines, one flavonoid, four nucleotides and their derivatives, one carbohydrate, one terpenoid and two other metabolites), and their relative contents peaked at stage S4.

### Screening of differential metabolites

To identify differentially accumulated metabolites, the metabolites in the soapberry pericarp from eight growth stages were subjected to a pairwise comparison according to their relative contents. Supervised OPLS-DA models were constructed, and the internally cross-validated figures of merit are listed in Table S3. *R*^2^*X*, *R*^2^*Y*, and Q^2^ of the fitting equations were all greater than 0.5, and the differences among the parameters were small (Table S3), indicating that the fitting equations were reliable. According to OPLS-DA score plots (Fig. S1), all samples in different groups were located in 95% confidence regions, and could be distinguished by OPLS models, indicating that there were significant differences between groups. Moreover, samples in the same group were concentrated, indicating good repeatability. As shown on the OPLS-DA permutation test (Fig. S2), all Q^2^ points were lower than the original Q^2^ points on the right. Q^2^ was less than 0, and the regression lines of R^2^ and Q^2^ crossed with the abscissa or were less than 0, demonstrating that these OPLS-DA models were reliable and effective^32^.

**Figure 4:**
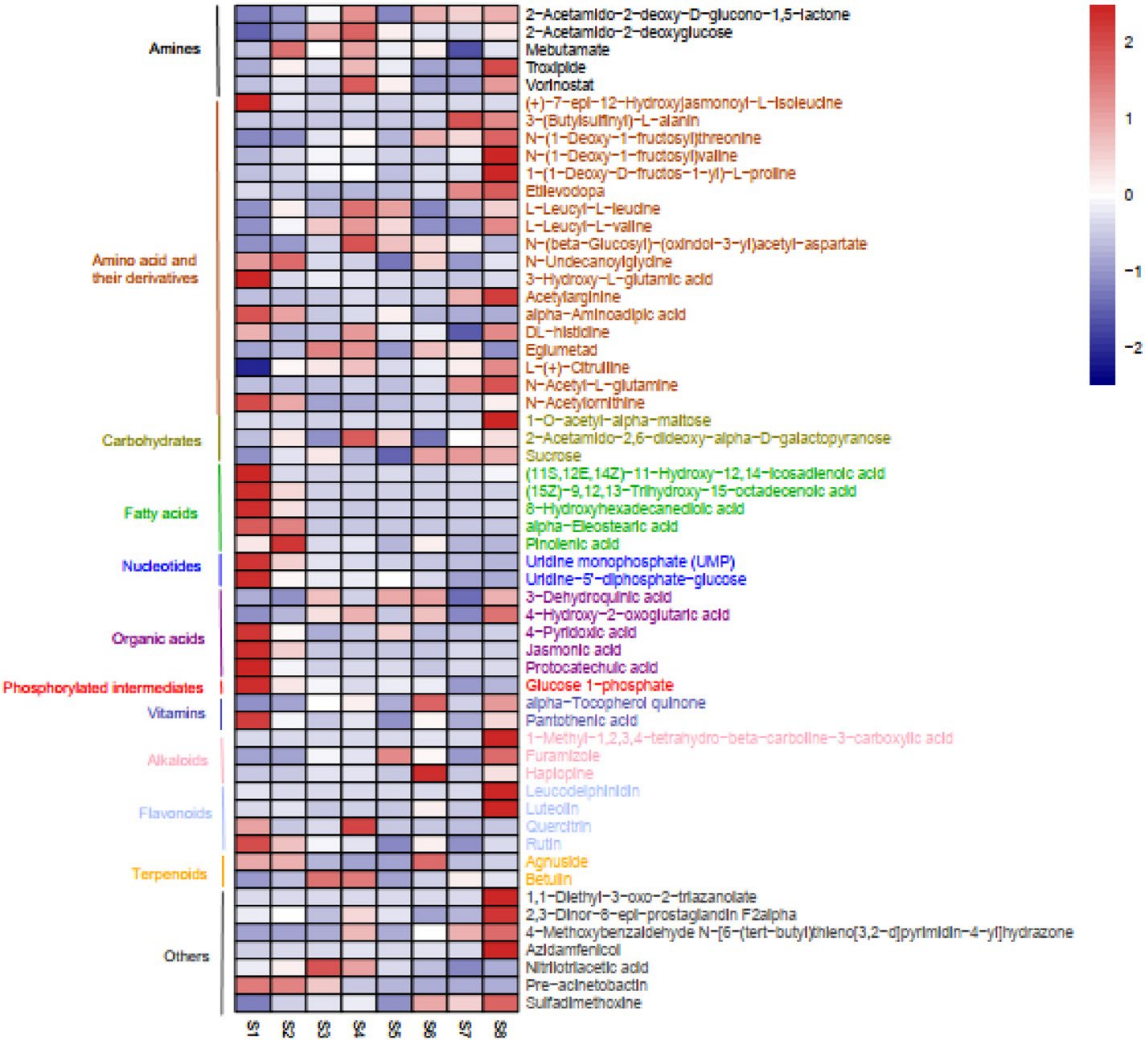
Heatmap of the 57 differential metabolites.

Based on the comparison, with VIP > 1, FDR < 0.05, and FC > 3 or < 0.33 as the threshold, 57 differential metabolites were identified (Table S4), including five amines, 18 amino acids and their derivatives, three carbohydrates, five fatty acids, two nucleotides, five organic acids, one phosphorylated intermediate, two vitamins, three alkaloids, four flavonoids, two terpenoids, and seven other metabolites. The average number of differential metabolites was five, and it ranged from zero (S2 vs. S8 and S3 vs. S7) to 18 (S1 vs. S6) (Table S5). Out of these metabolites, five amino acid and their derivatives [(+)-7-epi-12-hydroxyjasmonoyl-l-isoleucine, *N*-undecanoylglycine, 3-hydroxy-l-glutamic acid, alpha-aminoadipic acid, and *N*-acetylornithine], five fatty acids [(11S,12E,14Z)-11-hydroxy-12,14-icosadienoic acid, (15Z)-9,12,13-trihydroxy-15-octadecenoic acid, 8-hydroxyhexadecanedioic acid, alpha-eleostearic acid, and pinolenic acid], two nucleotides (uridine monophosphate and uridine-5′-diphosphate-glucose), three organic acids (4-pyridoxic acid, jasmonic acid, and protocatechuic acid), one phosphorylated intermediate (glucose 1-phosphate), one vitamin (pantothenic acid), one flavonoid (rutin), and one other metabolite (pre-acinetobactin) exhibited the largest relative content at stage S1 or S2, after which the content declined in a developing-dependent manner (Fig. 4).

In contrast, seven amino acids and their derivatives, two carbohydrates, two alkaloids, two flavonoids and five other metabolites, including 3-(butylsulfinyl)-l-alanin, *N*-(1-deoxy-1-fructosyl)threonine, *N*-(1-deoxy-1-fructosyl)valine, 1-(1-deoxy-d-fructos-1-yl)-l-proline, etilevodopa, acetylarginine, *N*-acetyl-l-glutamine, 1-*O*-acetyl-alpha-maltose, sucrose, furamizole, 1-methyl-1,2,3,4-tetrahydro-beta-carboline-3-carboxylic acid, leucodelphinidin, luteolin, 1,1-diethyl-3-oxo-2-triazanolate, 2,3-dinor-8-epi-prostaglandin f2alpha, 4-methoxybenzaldehyde *N*-[6-(tert-butyl)thieno[3,2-d]pyrimidin-4-yl]hydrazone, azidamfenicol, and sulfadimethoxine, gradually increased after stage S5 and peaked at stage S8 (Fig. 4).

After stage S4, three amines (2-acetamido-2-deoxy-d-glucono-1,5-lactone, troxipide, vorinostat), four amino acids and their derivatives (l-leucyl-l-leucine, l-leucyl-l-valine, dl-histidine, l-(+)-citrulline) and one carbohydrate (2-acetamido-2,6-dideoxy-alpha-d-galactopyranose) gradually decreased up to stage S7, and then largely increased at stage S8 (Fig. 4). In addition, the contents of several more important amines, amino acid and their derivatives, flavonoids, terpenoids, and other metabolites were relatively high at stage S4, and then gradually decreased during pericarp developing; specifically, 2-acetamido-2-deoxyglucose, mebutamate, *N*-(beta-glucosyl)-(oxindole-3-yl)acetyl-aspartate, eglumetad, quercitrin, betulin, and nitrilotriacetic acid (Fig. 4).

Two organic acids (3-dehydroquinic acid, 4-hydroxy-2-oxoglutaric acid), one vitamin (alpha-tocopherol quinone), and one alkaloid (haplopine) decreased from stage S6 to stage S7, but increased at stage S8 (Fig. 4). Additionally, agnuside had a high accumulation level in stages S1, S2 and S6 (Fig. 4).

### KEGG pathway analysis of the differential metabolites

**Figure 5:**
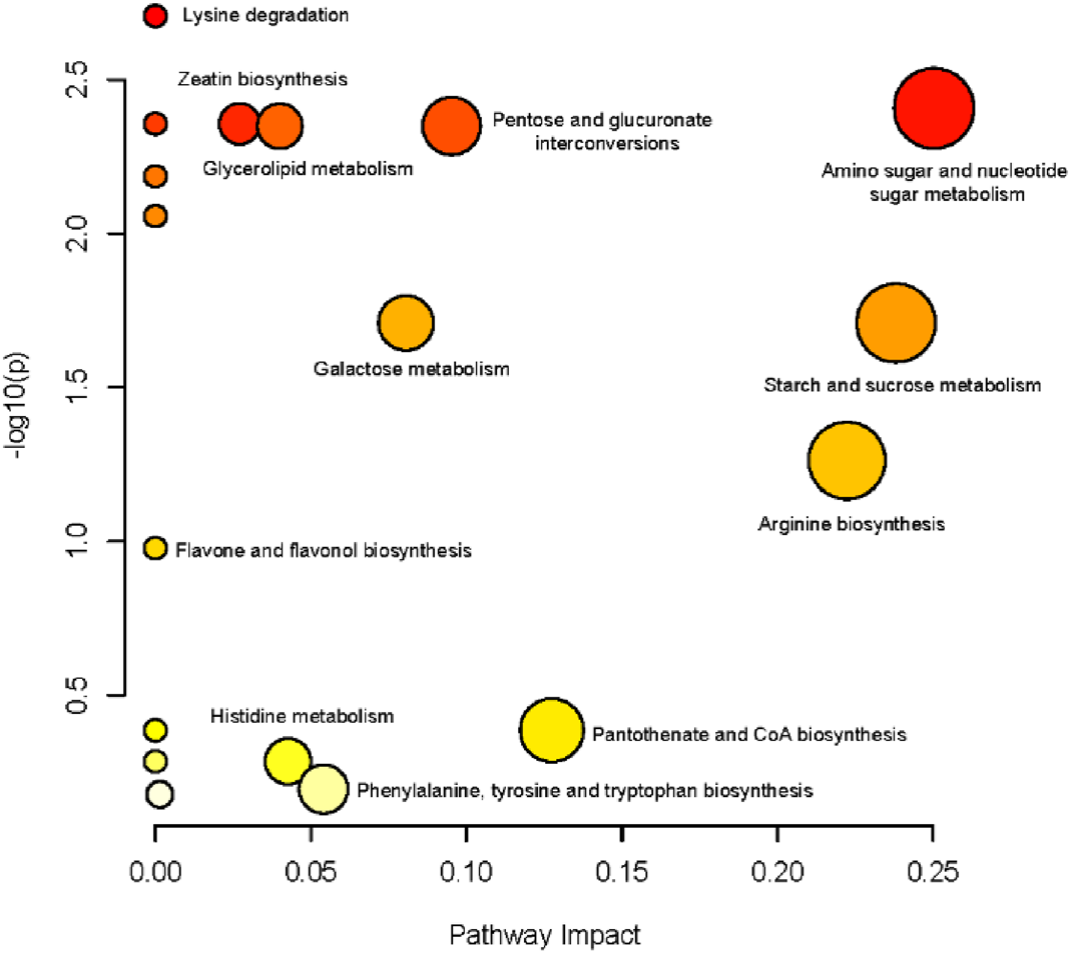
KEGG metabolic pathway classification of the differential metabolites. The node color is based on the p value, white and red colors indicate lower or higher p value, respectively. The node radius is based on the pathway impact value, small and large radius indicate lower or higher impact value, respectively.

To better elucidate the biological functions of the differential metabolites, a pathway analysis was performed by comparing the metabolites with the KEGG reference pathway. As expected, 15 of the 57 differential metabolites were involved in 18 different pathways (Table S6). Figure 5 shows the influence factors of metabolic pathways. Five pathways, namely lysine degradation, amino sugar and nucleotide sugar metabolism, zeatin biosynthesis, ascorbate and aldarate metabolism, and pentose and glucuronate interconversions, were identified as the most relevant.

## Discussion

LC–MS is currently the most widely used method for determining metabolic phenotypes via both untargeted and targeted analyses^33^. Metabolomic studies are often performed using RP chromatography (particularly C18) because of its robust and reproducible separation characteristics, as well as the coverage of a wide range of metabolites. Nevertheless, most biological matrices contain abundant polar metabolites that cannot be retained in RP stationary phases. Recently, HILIC has appeared to be the best LC approach to complement RP chromatography for the separation of polar compounds^34,35^. RP-based methods are used for medium to nonpolar metabolites, while HILIC is employed for more polar metabolites that are not well retained in RP systems^36^. In recent years, the application of HILIC in non-targeted metabolomics has increased^37,38^. Using two independent separation assays, such as RPLC and HILIC, coupled to a UHPLC-HRMS can expand the metabolic profiling coverage^28^. Here, we conducted a non-targeted metabolomic study of the soapberry pericarp during fruit growth based on UHPLC-HRMS with a C18 column and a HILIC column. After pre-processing, 1790 features were extracted on a C18 column in negative mode, while 13,000 features (5000 in negative mode and 8000 in positive mode) were extracted on the HILIC column, suggesting that the HILIC column was better than the C18 column in the non-targeted metabolomics analysis of the soapberry pericarp. This may have been because of the presence of more polar compounds in the pericarp, as we putatively annotated 34 amino acids and their derivatives, 12 organic acids and 10 fatty acids. In addition, HILIC can be coupled to MS, especially in the electrospray ionization (ESI) mode, thereby achieving high compatibility and sensitivity^26^. The positive mode using a C18 column in our study failed to analyze any of the metabolites, and this might have been caused by the unsuitable mobile phase and gradient elution conditions used in LC. Additionally, only Zhang et al.^39^ and Ling^40^ respectively used HPLC-electrospray ionization and quadrupole time-of-flight mass spectrometry (HPLC-ESI-QTOF-MS/MS) and UHPLC-linear ion trap-Orbitrap high-resolution mass spectrometry (UHPLC-LTQ Orbitrap MS) to carry out qualitative analysis of triterpenoid saponins in the soapberry pericarp. However, we did not find triterpenoid saponins in our non-targeted metabolomics data using both RPLC-HRMS and HILIC-HRMS assays. This may have been due to differences in the sample preparation and analytical methods. Furthermore, the LC conditions used in this study were also different from those of the two studies mentioned above. The reason may be the insufficient reports about soapberry saponins, lack of MS spectra information in various online databases, and unavailable authentic standards for LC. None of the currently available LC separation modes offer the ability to monitor all types of metabolites encountered in metabotyping studies^33^. In the future, serial coupling of RPLC and HILIC may be used to obtain the comprehensive metabolome coverage and to study the non-targeted metabolomics of soapberry pericarp. It may also be used to optimize sample preparation, chromatographic conditions, and MS parameters^33,36,41^.

PCA score plots indicated a clear separation of groups among the pericarps at different stages of soapberry fruit growth, demonstrating that the data in different stages were significantly different and that changes in the metabolic spectrum occurred in the process of fruit growth. In previous studies, metabolites in the pericarps of litchi^42^, pineapple^17^, banana^19^, and citrus^43^ mainly included sugars, amino acids, organic acids, alcohols, fatty acids, and others. Similar results were observed in the current study. We identified 34 amino acids and their derivatives, 12 organic acids, 10 fatty acids, nine amines, six flavonoids, six nucleotides and their derivatives, five alkaloids, four carbohydrates, four terpenoids, and 21 other metabolites in the soapberry pericarp. However, the main components of these metabolite types in the pericarps of different plants may be different. Based on the HCA, soapberry pericarp growth was clustered into three major stages, including S1–S2 stages (early fruit development), S3–S5 stages (late fruit development), and S6–S8 stages (fruit ripening). Additionally, the 111 annotated metabolites in the present study showed three different patterns of accumulation in the pericarp at eight different growth stages, which highlighted a clear metabolic shift among the early fruit development stages (S1–S2) to late fruit development stages (S3–S5) and then to fruit ripening stages (S6–S8). Contributing metabolites related to fruit growth process could be obtained from the VIP scores, FC values and FDR. With VIP > 1, FDR < 0.05, and FC > 3 or < 0.33 as the threshold, a total of 57 key metabolites were obtained.

Out of these 57 more important metabolites, the levels of five amino acid and their derivatives, four fatty acids, two nucleotides, three organic acids, one phosphorylated intermediate, one vitamin, and one flavonoid were high at stage S1 and significantly decreased at stage S2. These metabolites may play an important role in the early fruit development stages of soapberry pericarp. Among them, *N*-acetylornithine was involved in arginine biosynthesis; jasmonic acid participated in the pathway of alpha-linolenic acid metabolism; pantothenic acid was involved in the pathway of pantothenate and CoA biosynthesis and beta-alanine metabolism. In addition, alpha-aminoadipic acid, uridine-5′-diphosphate glucose, and glucose 1-phosphate were intermediate in the formation of lysine, biosynthesis of polysaccharides, degradation of glycolysis or glycogen, respectively^44,45^. Furthermore, jasmonic acid is a hormone naturally produced in plants; pantothenic acid is a growth factor that is essential for various metabolic functions, including the metabolism of carbohydrates, proteins, and fatty acids^44^. These two key metabolites have high content in the early stage of fruit development, which may promote the growth of soapberry pericarp, but the specific mechanism needs to be further studied. 8-Hydroxyhexadecanedioic acid is a component of various plant cutins^46^, which may play a protective role in the soapberry fruit. Additionally, the level of rutin was higher in the early stage of fruit development in our study, which may help soapberry fruit better resist ultraviolet radiation and other adverse environments^47^.

The content of five key amines, six amino acid and their derivatives, one carbohydrate, one flavonoid and one terpenoid noticeably changed at stage S4 of soapberry pericarps. With the changes of these metabolites, we also found that the diameter of soapberry fruit used in this study increased from 70% of the biggest fruit size at stage S3 to 90% of the biggest fruit size at stage S5. Changes in the content of these metabolites might probably affect the fruit development. Among them, some metabolites are involved in the important metabolic pathways related to the development of soapberry pericarp. For example, 2-acetamido-2-deoxyglucose was involved in amino sugar and nucleotide sugar metabolism; l-(+)-citrulline and dl-histidine participated in the pathway of arginine biosynthesis, histidine metabolism and aminoacyl-tRNA biosynthesis. In addition, these significant amino acids may be the main building blocks of proteins in soapberry pericarps^48^. Surprisingly, quercitrin accumulated at higher levels in stage S4 than other stages, suggesting that the pericarps had a strong ability of scavenging reactive oxygen species and could resist ultraviolet radiation^47^. Betulin also has a higher content at stage S4, and it exhibits a wide spectrum of biological and pharmacological properties, such as anti-HIV, anti-inflammatory, and anti-cancer^49^. This triterpene is a precursor of the triterpenoid saponins of soapberry. As we know, a lupine-type triterpenoid saponin, betulinic acid 3-*O*-β-d-xylopyranosyl-(1 → 3)-α-l-rhamnopyranosyl-(1 → 2)-α-l-arabinopyranoside, isolated by Hu et al.^6^ from the soapberry pericarp is derived from betulin. Although triterpenoid saponins have not been identified in this study, targeted metabolomics may be used to study triterpenoid saponins in the future.

Contrary to the early stages of development, which are characterized by high levels of key fatty acids, nucleotides, organic acids, and phosphorylated intermediates, in the ripening stages, the majority of these compounds decreased. However, the contents of most key amino acid and their derivatives, carbohydrates, alkaloids, flavonoids and other metabolites were high during ripening, especially at stage S8. With regard to sucrose, very low levels were found at the fruit development stages (S1–S5). Then, sucrose content in the pericarps highly increased from S5 to S6 and remained at high levels during fruit ripening (stage S6–S8). Similar results were observed in the pericarp of pineapple during ripening^17^. Sucrose is considered to be the source of sweetness in food and is generally used as the standard solution for sweetness^50^. Moreover, it regulates the development and ripening of fruit^51^. Studies have shown that sucrose accumulation might induce the expression of key enzymes in the abscisic acid (ABA) hormone pathway, thus promoting non-climacteric fruit ripening through ABA hormone^52^. Therefore, sucrose may modulate the ripening process in soapberry fruit. In addition, luteolin is a more powerful reactive oxygen species scavenger than other flavonoids^47^. The high accumulation of luteolin during fruit ripening may be related to environmental adaptation (such as ultraviolet radiation).

Interestingly, several bioactive metabolites were accumulated at high levels at stage S8, including troxipide, vorinostat, etilevodopa, dl-histidine, l-(+)-citrulline, alpha-tocopherol quinone, furamizole, and luteolin. For example, troxipide has inhibitory effects on human neutrophil migration and activation induced by various stimulants^53^; vorinostat is an oral histone deacetylase inhibitor with antineoplastic activity, which is approved for use in refractory or relapsed cutaneous T-cell lymphoma^54^; etilevodopa is a tyrosine derivative that can be used to treat Parkinson’s disease^44^; l-(+)-citrulline is a non-essential amino acid, whereas dl-histidine is an essential amino acid; alpha-tocopherol quinone can decrease androgen receptor protein and transcript levels in prostate cancer cells^36^; furamizole is a nitrofuran derivative with strong antibacterial activity^55,56^; luteolin is a naturally occurring flavonoid with potential antioxidant, anti-inflammatory, apoptosis-inducing, and chemopreventive activities^44^. Specifically, four differential flavonoids (namely, quercitrin, leucodelphinidin, rutin, and luteolin) were involved in flavonoid biosynthesis and flavone and flavonol biosynthesis in the current. Researchers have studied flavonoids from the leaves and stem bark of soapberry^57,58^. Therefore, flavonoids may play an important role in soapberry. Hence, the distribution, composition, bioactivity, and biosynthesis of flavonoids in soapberry should be investigated in the future.

Harmful compounds, such as 5′-*N*-ethylcarboxamidoadenosine, methyldopa and nitrilotriacetic acid, were also found in our metabolome data. According to the PubChem database, 5′-*N*-ethylcarboxamidoadenosine is of hazard class and category code (s) “Acute Tox. 2,” with an H300 hazard statement, indicating that it can be fatal, if swallowed^44^. In addition, methyldopa can cause acute and chronic liver injury, with a possibility of being fatal, and has a reproductive toxicity of H361 (100%), suspected of damaging fertility or an unborn child^44^. Nitrilotriacetic acid, which is considered to be a human carcinogen (NCI05), can also irritate the skin, eyes, and respiratory tract, as well as cause kidney and bladder damage^44^. However, these compounds exhibit biological and pharmacological activities. For instance, 5′-*N*-ethylcarboxamidoadenosine is an antineoplastic agent and a vasodilator agent, and methyldopa has antihypertensive activity^44^. Nitrilotriacetic acid is mainly used as a chelating and eluting agent and is found in laundry detergents. In addition, these were l-(+)-tartaric acid and 2-acrylamido-2-methyl-1-propane sulfonic acid, which are corrosive, were present in the pericarp of soapberry. l-(+)-tartaric acid is often used as an antioxidant food additive. However, at high doses, this agent acts as a muscle toxin by inhibiting the production of malic acid, which can cause paralysis and potentially lead to death^44^. 2-Acrylamido-2-methyl-1-propane sulfonic acid is a Food and Drug Administration-approved compound, for use in polymer components of food-contact paper and board adhesive, but if not used properly, its usage can lead to severe skin burns and eye damage^44^. These results verified the theory that “the pericarps of soapberry can be used as medicine, but have a small toxicity on people”, as recorded in the Compendium of Materia Medica, but the specific mechanism requires further study. Therefore, these factors should be considered in the future, and it is not recommended to use this pericarp without prescription.

The soapberry pericarp has high economic value. Our study elucidated the changes in metabolite contents of pericarp during soapberry fruit growth, which could be helpful in determining the optimal harvest stage of soapberry fruit. According to our investigation of fruit morphology, the soapberry fruit grows rapidly during the early stage, and can grow to its maximum size at the beginning of the maturity stage (S6), and the pericarp volume is also the largest at this time^13^. Although most key amines, fatty acids, nucleotides, organic acids, and phosphorylated intermediates highly accumulated in the pericarp at the early ovary development stage (S1) and 80% of the biggest fruit size stage (S4), the first five stages were not suitable for fruit harvesting, considering the maximization of economic benefits. However, during the later stage of fruit growth, the levels of several bioactive metabolites (i.e., troxipide, vorinostat, furamizole, alpha-tocopherol quinone, luteolin, and sucrose) were rapidly accumulated at stage S8 (fully developed and mature stage), so stage S8 was the most suitable stage for fruit harvesting, and the pericarp collected at this time had the highest utilization value.

## Conclusion

To our knowledge, this is the first study to use a non-targeted UHPLC-HRMS approach to investigate the metabolite profile of the soapberry pericarp at eight different fruit growth stages. The metabolome coverage of the HILIC column was higher than that of the C18 column, suggesting that HILIC may be more suitable for non-targeted metabolomic analysis of the soapberry pericarp. Moreover, 111 metabolites were putatively annotated. Principal component analysis and hierarchical clustering analysis of pericarp metabolic composition revealed clear metabolic shifts from early (S1–S2) to late (S3–S5) development stages to fruit ripening stages (S6–S8). Furthermore, pairwise comparison identified 57 differential metabolites that were involved in 18 KEGG pathways. Early fruit development stages (S1–S2) were characterized by high levels of key fatty acids, nucleotides, organic acids, phosphorylated intermediates, whereas several bioactive and valuable metabolites (i.e., troxipide, vorinostat, furamizole, alpha-tocopherol quinone, luteolin, and sucrose) were highly accumulated during fruit ripening (S6–S8). S8 (fully developed and mature stage) was the most suitable stage for fruit harvesting to utilize the pericarp. This study contributes to the study of biological activity or the traditional medicinal use of plants by offering a comprehensive view of the variations in the soapberry pericarp metabolome during fruit development. It can also serve as a guide for harvesting, processing, and application and can provide a conceptual framework for further studies on the biosynthesis of the main metabolites of the soapberry pericarp.

## Supplementary Information

Supplementary Figures.

Supplementary Tables.
