# Supplementary material for: Metabolomics analysis of the soapberry (Sapindus mukorossi Gaertn.) pericarp during fruit development and ripening based on UHPLC-HRMS
Source: Sci Rep. 2021 Jun 2;11:11657. doi: 10.1038/s41598-021-91143-0 (PMC8172880; doi:10.1038/s41598-021-91143-0)
Supplement: Supplementary file 1 — Supplementary Figures. [file 41598_2021_91143_MOESM1_ESM.docx]

**Metabolomics analysis of the soapberry (*Sapindus mukorossi* Gaertn.) pericarp during fruit development and ripening based on UHPLC-HRMS**

Yuanyuan Xu^1,2,3^, Gao Yuan^4^, Zhong Chen^1,2,3,5^, Guochun Zhao^1,2,3^, Jiming Liu^1,2,3^, Xin Wang^1,2,3^, Shilun Gao^1,2,3^, Duanguang Zhang^6^, Liming Jia^1,2,3,⁎^

^1^ Key Laboratory of Silviculture and Conservation of the Ministry of Education, College of Forestry, Beijing Forestry University, Beijing 100083, China

^2^ National Energy R&D Center for Non-food Biamass, Beijing Forestry University, Beijing 100083, China

^3^ National Innovation Alliance of Sapindus Industry, Beijing Forestry University, Beijing 100083, China

^4^ Planning and Design Institute of Forest Products Industry, National Forestry and Grassland Administration, Beijing 100010, China

^5^ Beijing Advanced Innovation Center for Tree Breeding by Molecular Design, Beijing Forestry University, Beijing 100083, China

^6^ Yuanhua Forestry Biological Technology Co., Ltd., Sanming, Fujian 650216, China

These authors contributed equally: Yuanyuan Xu, Yuan Gao.

^⁎^Corresponding to: Professor Liming Jia, Ph.D.

College of Forestry, Beijing Forestry University, 35 E Qinghua Road, Beijing 100083, China

E-mail address: jlm@bjfu.edu.cn (L. Jia).

Tel.: +86-010-6233-7055

E-mail address of other authors: yuanyuanxu_2016@163.com (Y. Xu); [gaoyuan@bjfu.edu.cn](mailto:gaoyuan@bjfu.edu.cn) (Y. Gao); sharazhonger@126.com (Z. Chen); zhaoguochun1122@126.com (G. Zhao); 18289143108@163.com (J. Liu); [1796850445@qq.com](mailto:1796850445@qq.com) (X. Wang)；gaoshilun7@sohu.com (S. Gao); [774629608@qq.com](mailto:774629608@qq.com) (D. Zhang).

**Supplemental file 2:**


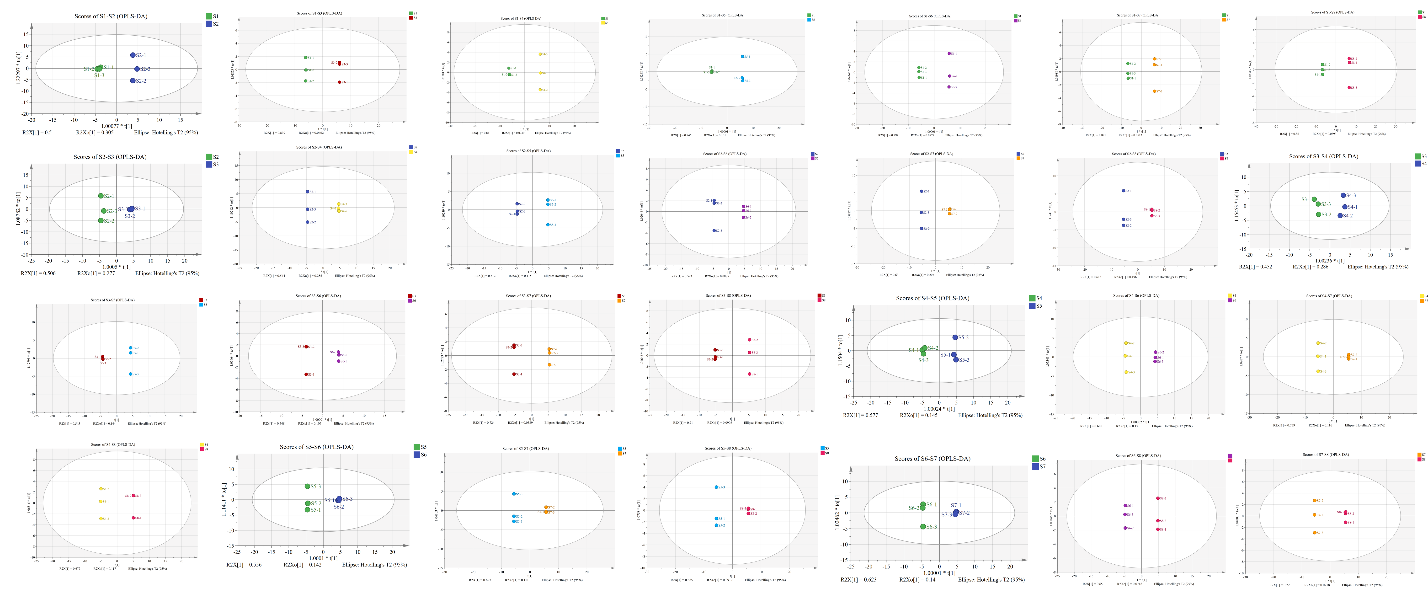


Fig. S1 OPLS-DA score plot


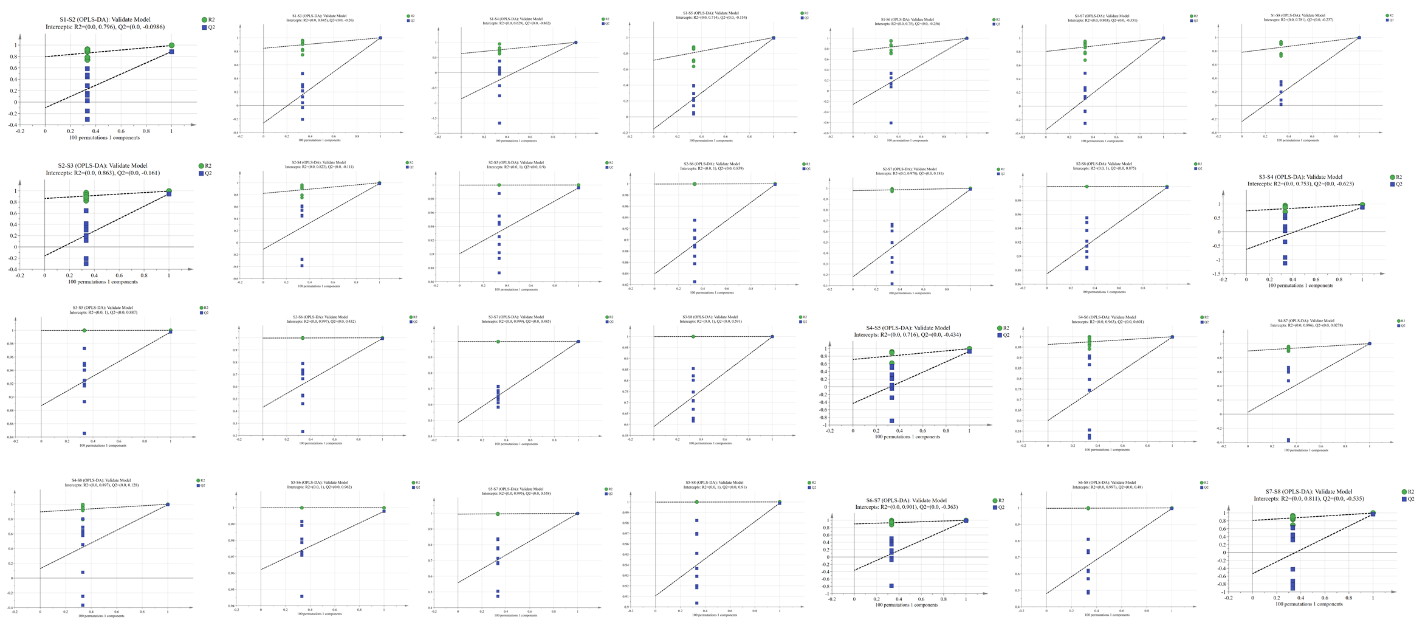


Fig. S2 OPLS-DA permutation test graph


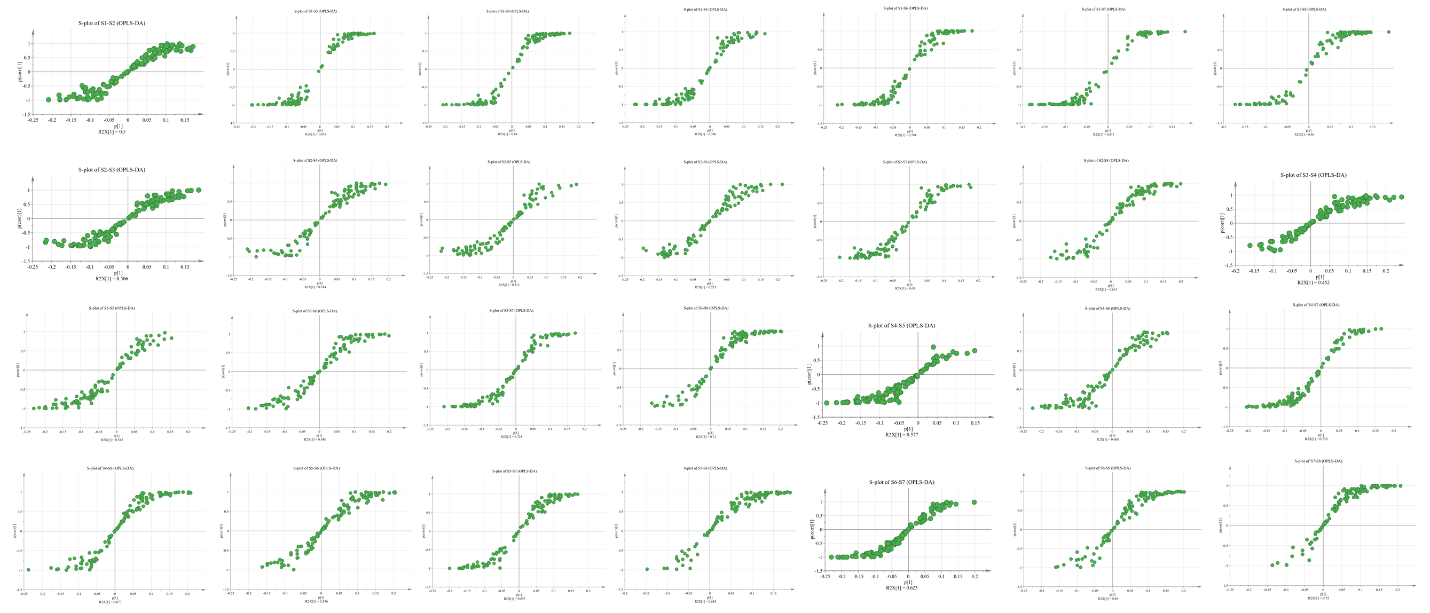


Fig. S3 OPLS-DA S-plot graph
